# Diagnostic accuracy of a molecular-based point-of-care test for rapid detection of respiratory syncytial virus in children admitted to high-dependency care in Nepal

**DOI:** 10.7189/jogh.16.04191

**Published:** 2026-08-28

**Authors:** Arun Kumar Sharma, Hari Prasad Kattel, Ram Hari Chapagain, Rupesh Shrestha, Prakash Joshi, Naresh Bahadur Khadka, Sangita Sharma, Yvette Löwensteyn, Neele Rave, Mattheus Cornelis Viveen, Louis Bont

**Affiliations:** 1 Department of Paediatrics, Maharajgunj Medical Campus, Tribhuvan University, Kathmandu, Nepal; 2 Department of Microbiology, Maharajgunj Medical Campus, Tribhuvan University, Kathmandu, Nepal; 3 Department of Paediatric Medicine, Kanti Children’s Hospital, Kathmandu, Nepal; 4 University Medical Centre Utrecht, Utrecht, the Netherlands

## Abstract

**Background:**

Rapid respiratory syncytial virus (RSV) diagnostics are largely unavailable in low-resource settings. We compared the diagnostic accuracy of a molecular-based point-of-care test (POCT) with standard reverse transcription polymerase chain reaction (RT-PCR) in children admitted to high-dependency care.

**Methods:**

We analysed data from a larger study (RSV GOLD – ICU Network study) in two Nepali tertiary hospitals over two respiratory seasons. Children aged ≥4 days and <2 years admitted to high-dependency units were tested for RSV using POCT at admission. We calculated sensitivity, specificity, and predictive values of POCT against laboratory RT-PCR on stored samples as the gold standard.

**Results:**

We analysed 124 randomly selected stored samples, of whom 70 (56.5%) tested positive with POCT and 74 (59.7%) with RT-PCR. The median cycle threshold (CT) value was 29.1 (interquartile range = 25.1–33.4). All cases with false-negative POCT had low viral loads (CT value >31). The sensitivity (93.2%; 95% confidence interval (CI) = 86.0, 97.5), specificity (98.0%; 95% CI = 91.5, 99.9), positive predictive values (98.6%; 95% CI = 93.9, 99.9), and negative predictive values (90.7%; 95% CI = 81.1, 96.6) of POCT for RSV infection were all satisfactory.

**Conclusions:**

We conducted the first prospective study showing excellent accuracy of molecular RSV POCT in a poor resource setting. Affordable molecular-based POCT in resource-constrained settings can improve care of children with acute respiratory infections and strengthen surveillance for vaccine effectiveness during rollout of RSV interventions.

Severe acute respiratory infections (SARIs) are major causes of hospital admissions and mortality worldwide in children aged <5 years [1]. Following the introduction of Pneumococcal and *Haemophilus influenzae* type b vaccines, viruses are now the dominant cause of SARI, with respiratory syncytial virus (RSV) being the most common pathogen in young children [2]. In 2019, RSV caused an estimated >33 million lower respiratory tract infections in young children, 95% of which occurred in low- and middle-income countries (LMICs) [3]. Almost one-third of all pneumonia cases in young children are attributed to RSV, and an even greater proportion of cases occur during the peak RSV season [2]. Against this backdrop, most clinicians in resource-limited settings prescribe antibiotics for pneumonia following Integrated Management of Childhood Illness guidelines [4], which may result in unnecessary antibiotic use in many children with viral pneumonias. This unnecessary antibiotic use remains problematic in LMICs and contributes to increasing antibiotic resistance [5].

The lack of diagnostic facilities for the quick identification of viruses responsible for childhood pneumonia is an important driver of unnecessary antibiotic use. Polymerase chain reaction (PCR)-based diagnosis is limited by availability, cost, and training requirements in many LMICs. Moreover, the lengthy turnaround time required for PCR results limits its use for decisions on antibiotic prescription in areas with high patient volumes. Easy availability of point-of-care tests (POCTs) for RSV identification, with rapid turnaround times, can significantly reduce high-volume unnecessary antibiotic prescriptions in these settings.

Molecular-based POCTs with high diagnostic accuracy have emerged in recent years but remain unavailable in LMICs and have not been well validated in these settings [6]. Challenges associated with the stability of devices and test materials at variable ambient temperatures may affect test validity in resource-limited settings [6]. Validation of the diagnostic accuracy of molecular-based POCTs in different settings can also contribute to the broader use in resource-limited areas, both for diagnostic purposes and for research in clinical trials. We aimed to compare a molecular-based POCT with standard reverse transcription PCR (RT-PCR) to determine the diagnostic accuracy of POCTs for identifying RSV in nasal swabs from children requiring high-dependency care in Nepal.

## METHODS

We conducted a sub-analysis of data collected during the RSV GOLD – ICU Network study [7,8]. The RSV GOLD – ICU Network study was a prospective observational study among children aged <2 years admitted to high-dependency care units in ten LMICs and the Netherlands during two RSV seasons. We analysed the data from Nepal, where the study was conducted at two referral hospitals in Kathmandu. The data collection, including the use of POCT and PCR analysis, was planned at study initiation; however, PCR was only performed in a subset of the stored samples after the preliminary analysis of the data was completed. Site-specific budgetary constraints, as well as the unavailability of RSV probes and primers at the time of conducting the main study, were the primary reasons for delayed PCR testing. The PCR testing was completed only after additional funding was secured, and the University Medical Centre (UMC) Utrecht provided test kits.

### Study setting

Study participants were recruited at the paediatric and neonatal high-dependency units during two RSV seasons at Kanti Children’s Hospital and Tribhuvan University Teaching Hospital between July 2021 and February 2023. The first season lasted from 19 July 2021 to 14 January 2022, and the second from 1 June 2022 to 19 February 2023. The researchers performed POCT at the bedside laboratory at the primary data collection time. They performed RT-PCR on stored samples at the Department of Microbiology, Tribhuvan University Teaching Hospital in April and May of 2024.

### Study population

**Figure 1:**
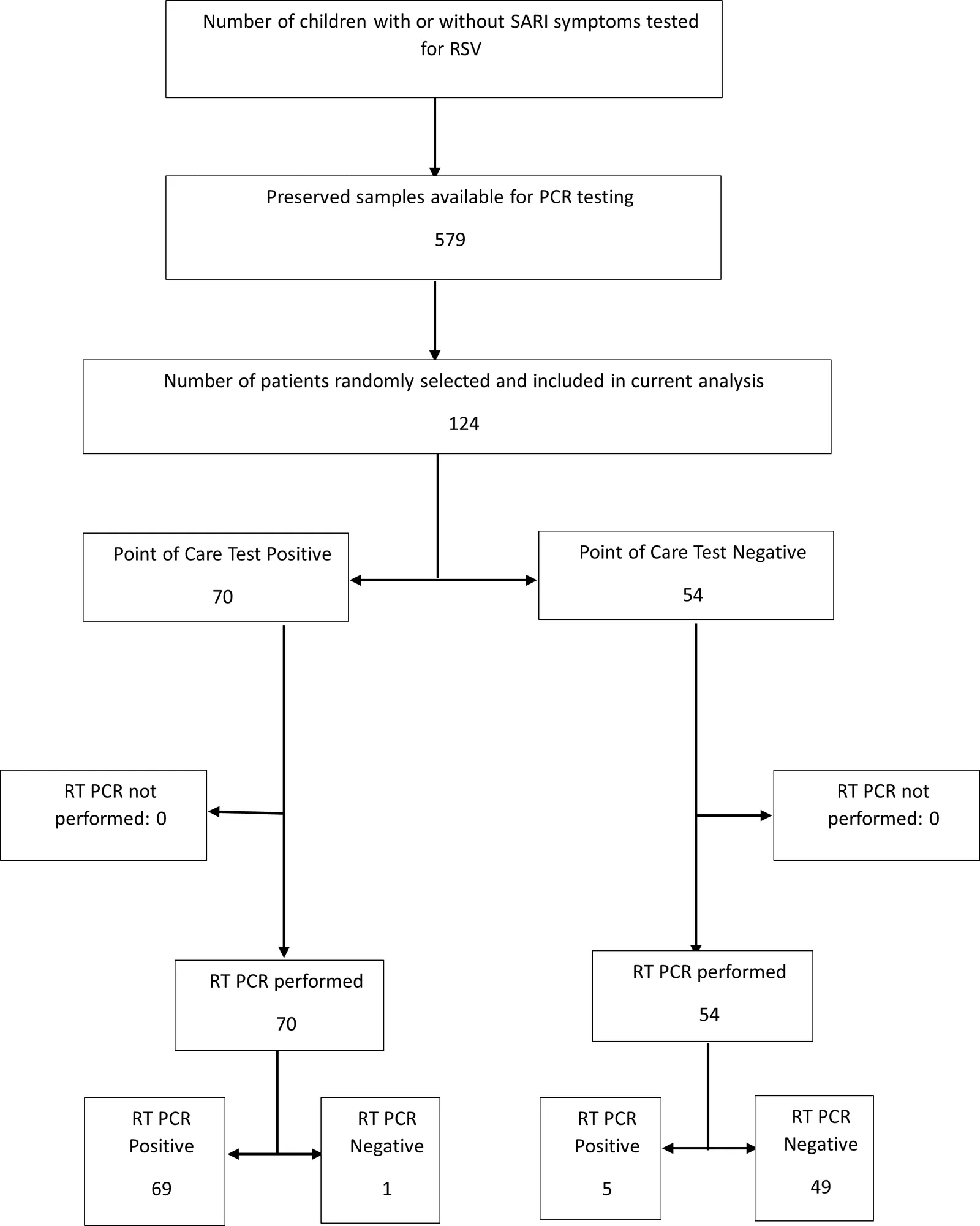
Study flowchart. PCR – polymerase chain reaction, RSV – respiratory syncytial virus, RT-PCR – reverse transcription polymerase chain reaction, SARI – severe acute respiratory infection

In the main study, children aged ≥4 days and <2 years admitted to high-dependency units of either hospital were eligible for participation. Children were grouped into two categories: the extended SARI (eSARI) group (Group A) (if they fulfilled the World Health Organization (WHO) eSARI criteria) and the non-eSARI group (Group B) (if they did not fulfil the eSARI criteria) [7,8]. For the sub-study, we randomly selected participants from Group A or B whose samples were preserved and available for analysis. Budget limitations precluded testing of all stored samples. We used a line list of participants with stored samples, along with their initial POCT results, to randomly select samples subjected to laboratory PCR (Text S1 in the **Online Supplementary Document**). We did the random selection using a computer-generated random number (**Figure 1**).

### Sample collection and storage

Nasal swabs were collected as soon as possible, but within 72 hours after admission to the hospital, using a Copan® flocked nasal swab, and trained study nurses immediately transferred the samples to Copan Universal Transport Medium [9]. The sample was either tested immediately using POCT or refrigerated at 2–8 °C and tested the following morning. RSV POCT was performed in all samples; influenza POCT was also included in children fulfilling eSARI criteria at admission. After completing the POCT, the remaining sample in the Universal Transport Medium was stored in cryovials at −40 °C in the Kanti Children’s Hospital research unit, and the freezer temperature was monitored by temperature logs twice a day. Nasal swab specimens were preserved for all children included in the main study in the 2021 season if consent was available for long-term storage. Due to physical constraints of the freezer space, only samples identified as RSV or influenza-positive by POCT were preserved during the 2022 season.

### Sample size

We based the minimum number of samples needed to be tested by RT-PCR for validation of ID NOW RSV POCT on the Buderer formula [10], assuming a minimum POCT sensitivity and specificity of 90% each. With an assumed RSV prevalence of 30% among all children admitted with eSARI [2], we estimated the number of samples required to be tested with PCR to achieve a precision of 5% at the 95% confidence interval (CI) at 116 and rounded it off to 122 to cover the loss of 5% of stored samples.

### Index test: RSV POCT

The RSV POCT was performed on the ID NOW platform manufactured by Abbott Diagnostics and stationed at the bedside laboratory. The Abbott ID Now instrument is intended for indoor use with a recommended storage and operating environmental temperature range of 15–30°C and relative humidity of 10–80% at an altitude of 0–2000 m [11]. Kathmandu stands at an altitude of 1400 m, and during the study, the average daily outdoor temperature was 17.9°C (standard deviation (SD) = 5.3; maximum = 25.6; minimum = 6.6°C), and the average daily outdoor humidity was 77.9 (SD = 9.8) [12]. The ID NOW instrument is a rapid molecular in vitro diagnostic test designed for POCTs. It can be used for the qualitative detection of RSV viral RNA and delivers results in ~ 15 minutes. The instrument utilises isothermal nucleic acid amplification technology to detect both RSV A and RSV B nucleic acids [9]. A laboratory assistant, trained for procedural quality control by the Dutch team and overseen by an in-house microbiologist, performed the ID NOW^TM^ RSV assay according to the manufacturer’s guidelines [9]. The instrument is equipped with internal procedural control for functionality of the amplification/detection process and reagents. Additionally, with each new test box of reagents, external positive and negative controls were performed according to the manufacturer’s recommendation using standard test kits provided with each box of reagents. For samples collected during routine working hours, the technician completed POCT upon receipt of samples; however, for samples refrigerated overnight, the samples were allowed to stand for 30–60 minutes to bring them back to room temperature before testing. The POCTs were reported as RSV positive or RSV negative according to the results provided by the instrument.

### Reference standard/RT-PCR

We evaluated the POCT RSV assay against a real-time RT-PCR as a gold standard and performed it at the PCR laboratory, Department of Microbiology, Tribhuvan University Teaching Hospital. Microbiologists and technicians in the PCR laboratory routinely perform RT-PCR assays for SARS-CoV-2, but not for RSV. We conducted RT-PCR following the RSV testing protocol developed by the UMC Utrecht. The laboratory personnel conducting the RT-PCR were blinded to the patients’ clinical characteristics and the POCT results and were only provided with a list of samples. Positive control of the PCR reaction was set up from a stock solution provided by the UMC Utrecht. The stock solution had RT-PCR quantification set up based on electron microscopy and counted RSV stocks [13].

We thawed the frozen samples to room temperature and used them for RNA extraction. We extracted RNA from 200 μL of the sample using the Invitrogen PureLink® RNA Mini Kit (Thermo Fisher Scientific) according to the manufacturer’s protocol. We eluted the nucleic acids in a volume of 50 μL, and used 5 μL of the elution for RT-PCR amplification. We separately detected RSV subgroups A and B by RT-PCR using fluorescently labelled RSV primers/probes directed at the highly conserved genomic regions of the N gene for both RSV subgroups (Table S1 in the **Online Supplementary Document**). Using a one-step fast-viral mastermix, we performed PCR on a Quantstudio 5 Applied Biosystems instrument used by the laboratory for routine molecular diagnostic testing. We ran PCR at a volume of 12.5 μL (1.25 μL of target primer/probe mix, 3.125 μL of one-step fast viral master mix, 3.125 μL of RNase-free water, and 5 μL of eluted sample) up to 45 cycles. We repeated RT-PCR results with inconclusive linear and logarithmic amplification plot results on the same platform in a similar protocol after repeated RNA extraction from preserved samples, and the repeat test results were recorded as the final PCR test result. We considered all results with a cycle threshold (CT) value of <45 to be positive.

Following the initial round of RT-PCR, we compared the results from POCT and RT-PCR to identify any discrepancies. For samples with discordant results, we repeated each test to verify the validity of the test results. A microbiologist at UMC Utrecht provided an expert review of the test results before the final disposition.

### Statistical analysis

We used SPSS, version 25 (IBM Corporation, Armonk, NY, USA) for all analyses. We presented baseline characteristics as proportions and appropriate summary measures. Using laboratory-based RT-PCR as the reference standard, we calculated the sensitivity, specificity, positive predictive value, and negative predictive value for RSV ID NOW. We performed a subgroup analysis separately for samples from children of different age groups and those with the presence or absence of WHO-defined eSARI criteria at the time of admission.

## RESULTS

During the study period, 1617 patients underwent POCT at admission, and 579 samples were preserved and available for analysis. We analysed 124 randomly selected samples, of which 91 were from season 1 and 33 from season 2. The median age was 100 days (interquartile range = 30–278), and most children (87.1%) had an eSARI diagnosis at admission (**Table 1**; Table S2 in the **Online Supplementary Document**). Most tested samples belonged to children with eSARI diagnosis who were generally younger than those without eSARI diagnosis (Table S3 in the **Online Supplementary Document**). In general, the POCT instrument delivered positive test results earlier than negative test results, with the average time being 10–20-minute for most tests.

**Table 1:** Characteristics of children included in the RT-PCR analysis*

	Total (n = 124)	POCT positive (n = 70)	POCT negative (n = 54)
**Child's age in months**			
<2	50 (40.3)	20(28.6)	30(55.6)
2–6	32 (25.8)	24(34.3)	8(14.8)
6–12	21 (16.9)	15(21.4)	6(11.1)
12–24	21 (16.9)	11(15.7)	10(18.5)
**Age in days, MD (IQR)**	100 (30–277)	132 (58–301)	43 (12–267)
**Birth weight (in kg), x̄ (SD) (n = 117)**	2.8 (0.53)	2.9 (0.53)	2.8 (0.53)
**Born preterm (<37 weeks) (n = 122)**	13 (10.5)	8 (14.8)	5 (7.3)
**Sex**			
Female	38 (30.6)	20 (28.5)	18 (33.3)
Male	86 (69.4)	50 (71.4)	36 (66.6)
**Nutritional status (WHO growth standards), x̄ (95% CI)**			
WFAZ	−1.4 (−1.7, −1.1)	−1.1	−1.9
HFAZ	−1.2 (−1.5, −0.9)	−1.0	−1.5
WFHZ	−0.8 (−1.2, −0.4)	−0.4	−1.4
**Breastfeeding status**			
Mixed breast and formula	61 (49.2)	29 (41.4)	32 (52.5)
Exclusively breastfed	57 (46.0)	37 (52.9)	20 (32.1)
Formula	3 (2.4)	2 (2.9)	1 (1.9)
Other	3 (2.4)	2 (2.9)	1 (1.9)
**Immunisation status (n = 123)**			
Fully immunised for the local schedule and age	60 (48.3)	42 (60)	18 (33.3)
Partially immunised for the local schedule and age	53 (43.0)	25 (35.7)	28 (51.8)
Not yet vaccinated	10 (8.1)	2 (2.8)	8 (14.8)
**Presence/absence of SARI at admission**			
Fulfilled eSARI criteria at diagnosis (Group A)	108 (87.1)	68 (97.1)	40 (74.1)
Did not fulfil eSARI criteria at diagnosis (Group B)	16 (12.9)	2 (2.9)	14 (25.9)
**SpO_2_ at admission, MD (IQR) (n = 105)**	88 (86–95)	88 (85–91)	94 (86–96)

CI – confidence interval, eSARI – extended severe acute respiratory infection, HFAZ – height-for-age Z score, IQR – interquartile range, MD – median, POCT – point-of-care test, RT-PCR – reverse transcription polymerase chain reaction, SARI – severe acute respiratory infection, SD – standard deviation, WFAZ – weight-for-age Z score, WFHZ – weight-for-height Z score, WHO – World Health Organization, x̄ – mean

*Values presented as n (%) unless specified otherwise.

All discordant test results were reconfirmed as valid in both test platforms. Of all samples, POCT was positive in 70 (56.5%) and RT-PCR in 74 (59.7%) of patients. Among PCR-positive samples, 46 (62.1%) were positive for RSV A, 26 (35.1%) for RSV B, and 2 (2.7%) for both. The median CT value of all PCR-positive samples was 29.1 (interquartile range = 25.1–33.4). Most patients in the 2021 season had RSV A infection; RSV B was detected in only one sample (0.11%) from that season, where it was co-detected with RSV A. Similarly, most patients had RSV B infection in the 2022 season; RSV A was detected in only 3 (9.1%) samples from that season, where it was co-detected with RSV B in one sample.

**Figure 2:**
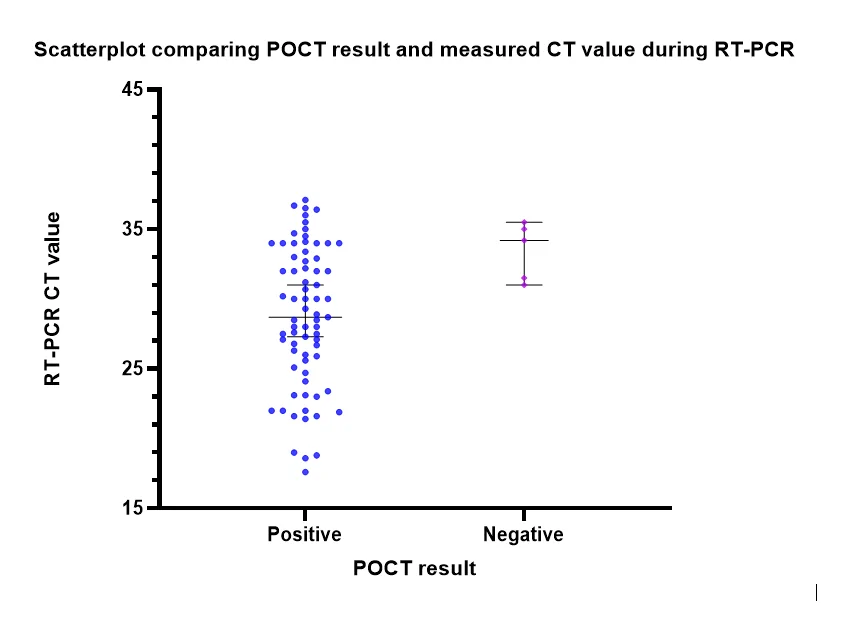
RSV RT-PCR CT value compared with POCT* results. *POCT instrument readout was positive or negative only; viral load or CT values are not reported. Each ‘blue circle legend’ or ‘purple diamond legend’ represents a single data point in POCT-positive (blue) and POCT-negative (purple) samples, respectively. Black lines represent the median with 95% CI error bars on either side. CI – confidence interval, CT – cycle threshold, POCT – point of care test, RSV – respiratory syncytial virus, RT-PCR – reverse transcription polymerase chain reaction.

There were six discordant test results between the POCT and laboratory RT-PCR. Only one sample tested positive by POCT was identified as negative by RT-PCR. Although the POCT device platform used molecular techniques, output was provided as a positive or negative test result only, without CT values that precluded analysing the correlation between results of POCT and RT-PCR. However, all five samples that tested positive by RT-PCR but reported negative by POCT had a CT value of >30, and three (60%) of these samples had a CT value of >34, suggesting low viral load. (**Figure 2**; Table S4 in the **Online Supplementary Document**).

Among discordant results between the POCT and RT-PCR (Table S4 in the **Online Supplementary Document**), one sample was falsely labelled as positive, and five samples were falsely labelled as negative by POCT compared to the gold standard. All five samples labelled negative by POCT but identified as positive in RT-PCR were collected from children in the 2021 season, which was predominated by RSV A. Sensitivity (93.2%; 95% CI = 86.0, 97.5), specificity (98.0%; 95% CI = 91.5, 99.9), positive predictive value (98.6%; 95% CI = 93.9, 99.9), and negative predictive value (90.7%; 95% CI = 81.1, 96.6) of POCT for any RSV infection were all satisfactory. We also performed subgroup analysis stratified by age and presence of eSARI criteria at admission, which showed that the POCT performed well in patients of all age groups with or without an eSARI diagnosis (**Table 2**). This analysis, however, was not based on the primary study objective, and no formal power calculations were made. Therefore, this result should be interpreted as exploratory.

**Table 2:** Diagnostic accuracy of RSV ID NOW POCT compared to RT-PCR as gold standard

	Total, n	TP, n	FP, n	FN, n	TN, n	Sensitivity, %	Specificity, %	PPV, %	NPV, %
All children*	124	69	1	5	49	93.2 (86.0, 97.5)	98.0 (91.5, 99.9)	98.6 (86.0, 97.5)	90.7 (86.0, 97.5)
**Age in months**									
<2	50	19	1	4	26	83.6	96.3	95	86.7
2–6	32	24	0	1	7	96	100	100	87.5
6–12	21	15	0	0	6	100	100	100	100
12–24	21	11	0	0	10	100	100	100	100
**SARI diagnosis at admission**									
SARI	108	67	1	4	36	94.4	97.3	98.5	90
Non-SARI	16	2	0	1	13	66.7	100	100	92.9

FN – false negative, FP – false positive, NPV – negative predictive value, POCT – point-of-care test, PPV – positive predictive value, RSV – respiratory syncytial virus, RT-PCR – reverse transcription polymerase chain reaction, SARI – severe acute respiratory infection, TN – true negative, TP – true positive

*Values for sensitivity, specificity, PPV, and NPV are presented as % (95% confidence interval).

## DISCUSSION

RSV diagnostic facilities are limited in Nepal, and rapid POCTs are not part of routine clinical care. In this setting, we found that RSV ID NOW POCTs based on molecular techniques demonstrated a high sensitivity of 93.2% (95% CI = 86.0, 97.5) and a specificity of 98.0% (95% CI = 91.5, 99.9) for the identification of RSV from nasal swabs in children admitted to high-dependency care. The test results were obtained in under 20 minutes.

The high diagnostic accuracy results identified align with the results reported in settings with different patient populations, environmental characteristics, and RSV disease epidemiology [14]. Molecular-based POC tests have reported sensitivity of 93–100% and specificity of 77–100% [14]. In general, rapid antigen detection tests for RSV are reportedly less sensitive than molecular tests [14–16]. Although traditional PCR remains the gold standard, it is severely limited by its turnaround time and complexity for rapid RSV diagnostics. The high sensitivity and specificity of molecular POCT for RSV in Nepali children highlight its applicability in diverse settings.

Despite the high cost and limited accessibility of RSV testing for routine use in most of the developing world, the implementation of rapid and accurate POC diagnostics holds considerable benefits. Such diagnostic facilities could potentially shorten or reduce hospitalisation, decrease the need for unnecessary diagnostic tests, allow the cohorting of young infants with RSV infections in hospitals to limit transmission, and reduce unnecessary antibiotic prescriptions for children who present with SARIs. Allen *et al.* reported significant cost savings in RSV care after using POCT in a high-resource setting [17]. The heavy acute respiratory infection burden in LMICs could translate to even greater economic benefits for both out-of-pocket and public health system healthcare expenses. Unnecessary antimicrobial prescriptions for all-cause acute respiratory infections are particularly common in LMICs and increase the cost of care and potentiate the emergence of antimicrobial resistance. Evidence is accumulating for the use of POCT for reducing this unnecessary antibiotic use [18]. We did not aim to directly evaluate the impact of POCT on reducing antibiotic prescriptions, hospitalisation rates, quality of care, or the economic burden for RSV-related hospitalisations. However, the good diagnostic accuracy of POCT we identified suggests potential benefits of deployment of this diagnostic modality in LMIC settings in reducing inappropriate antibiotic use and improving RSV-related care. Moreover, the anticipated cost for the expansion of POC diagnosis should be considerably lower than that of establishing a molecular laboratory facility.

We found variation in RSV subtypes during two respiratory seasons. These results raise the possibility that there may be year-over-year changes in circulating RSV subtypes among young Nepali children. Although we randomly selected the samples for RT-PCR testing because the POC output did not specify RSV subtypes, several limitations must be acknowledged when interpreting the results. The seasonality data spanned only two years, the sample size was not particularly large, and we did not preserve all swabs in the second RSV season. However, yearly changes with alternating predominant RSV subtypes are globally well recognised [19]. Mean viral loads in RSV A and RSV B positive samples were not significantly different, and POCT performed well for both subtypes.

Several RSV interventions, including RSV vaccines and immunoglobulins, are being introduced and have the potential to significantly reduce RSV-related mortality and morbidity [20]. Concerns for incremental health care system costs to incorporate these interventions into the immunisation programme, along with the financial burden of *Haemophilus influenzae* type band *Streptococcus pneumoniae* vaccines for acute respiratory infection control, may alarm policymakers in LMICs. The expansion of rapid, accurate, molecular-based RSV diagnostics requiring limited technical skill can provide strong evidence of disease burden to policymakers to continue investing in RSV interventions. In addition, POCT, suitable for deployment as a surveillance tool in settings with limited access and laboratory capabilities, can play a significant role in case identification and management following new vaccine introduction. It can also enable more comprehensive evaluations of vaccine effectiveness in clinical trials and the national immunisation programmes [21]. Rapid diagnostic tests have proven to be useful in assessing vaccine effectiveness for other infectious diseases [21–24]. Our results suggest the potential application of molecular-based POCTs in routine clinical care for accurate early identification of RSV, particularly in settings such as Nepal and other LMICs where routine laboratory PCR is not available. However, the diagnostic accuracy of POCT for identification of RSV in nasal specimens in asymptomatic children and children presenting with non-respiratory symptoms should be considered carefully (Table S5 in the **Online Supplementary Document**).

We acknowledge several limitations in this analysis. First, we compared POCT against a single RT-PCR as a reference standard and divergent results were retested using the same target PCR reaction. Repeat PCR assay to assess reproducibility is generally preferred to be conducted on different days, by different operators, using different instruments and validated probe/primer targets for amplification. Differential yield of alternative RT-PCR results could suggest different test sensitivity and specificity for POCT than we observed. Second, we included children who were not primarily admitted with acute respiratory disease. It is expected that POCTs perform better for children with SARI during the RSV season, when patients with higher viral loads and with more severe symptoms are likely to be admitted. Conversely, RSV colonisation of the nasopharynx in children with minimal respiratory symptoms, and those who are already recovering, is likely to be associated with lower viral loads below the detection threshold of POCT, which may increase the risk of false-negative results. Although four POCT-negative and five PCR-positive children had a SARI diagnosis at admission, all samples had a CT value of >31, suggesting borderline or low viral load. Here, the primary SARI diagnosis could also be related to coinfection, as we did not evaluate other viruses. Third, although available data suggest that the POCT performed reasonably well across all age groups, the number of children within each age stratum was small, and we did not perform formal power calculations for stratified analysis, as this was not our primary objective. Consequently, this limits the generalisability of study results across different age groups. Lastly, we performed POCT at the time of sample collection and PCR after prolonged storage. Sample storage in low-resource settings is not always optimal due to power supply fluctuations and equipment malfunction. The study samples were stored at −40°C, as ultra-low freezers were not available. We avoided freeze-thaw cycles, but potential nucleic acid degeneration over time during storage and sample thawing at time points of testing could have resulted in lower viral detection rates compared to testing fresh specimens, limiting the generalisability of the study results across diverse settings [25].

## CONCLUSIONS

Rapid molecular-based POC tests can be useful for the rapid diagnosis of RSV in children admitted to high-dependency care during RSV season in Nepal and similar settings. Although the generalisability of our results is limited, low-cost, simple POCT platforms suitable for widespread use in resource-limited settings may have the potential to improve care of sick children by reducing financial burden, limiting unnecessary antibiotics, and preventing the emergence of antibiotic resistance. They may also support assessment of vaccine effectiveness during the rollout of RSV interventions anticipated in the coming years.

## Additional material


Online Supplementary Document
