# Supplementary material for: Diagnostic accuracy of a molecular-based point-of-care test for rapid detection of respiratory syncytial virus in children admitted to high-dependency care in Nepal
Source: J Glob Health. 2026 Aug 28;16:04191. doi: 10.7189/jogh.16.04191 (PMC13521485; doi:10.7189/jogh.16.04191)
Supplement: Supplementary file 1 [file jogh-16-04191-s001.pdf]

Supplement to: Sharma AK, Kattel HP, Chapagain RH, Shrestha R, Joshi P, Khadka NB, Sharma S, Löwensteyn Y, Rave N, Viveen MC, Bont L. Diagnostic accuracy of a molecular-based point-of-care test for rapid detection of respiratory syncytial virus in children admitted to high-dependency care in Nepal. J Glob Health. 2026;16:04191.

SUPPLEMENTARY MATERIAL

Text S1

PCR was performed on stored samples after completion of participant recruitment and primary analyses, as part of the RSV GOLD III–ICU Network study.

In the parent study (The RSV GOLD III – ICU Network study)[1,2], children admitted to high-dependency units were enrolled and classified based on the presence of the World Health Organization (WHO) extended Severe Acute Respiratory Infection (eSARI) criteria. Children who fulfilled the eSARI criteria were assigned to Group A, while those who did not fulfil the criteria were assigned to Group B.

The point-of-care ID NOW™ RSV test (Abbot) was performed at the bedside laboratory according to the instructions included in the package insert. The ID Now RSV assay on the POCT instrument provides a qualitative result and does not distinguish between RSV subtypes. A summary of POCT results, along with the number of samples tested in each group of children, is presented in Supplementary Table S1A.

During the 2021 respiratory season, specimens were stored for all children included in the main study if consent was available for long-term storage and a sufficient sample volume was available. However, the freezer space was inadequate for preservation of all samples in the 2022 respiratory season. RSV genotyping was also planned at the study initiation. However, this was subject to funding availability. To offset the deficiency of adequate freezer space for all samples and to ensure that all RSV positive samples got stored for future RSV genotyping if funds became available, only samples identified as RSV- or influenza-positive by POCT were preserved during the 2022 season.

A line list of participants with their initial POCT results was available at the time of laboratory PCR testing. We decided to use a balanced mix of stored samples in each study group based on POCT results for laboratory PCR. The line list identified participants from Group A and Group B, as well as RSV POCT-positive and POCT-

negative samples. We randomly selected a proportionate number of samples for laboratory PCR from each group based on a list of random numbers generated by a computer using an online random number generator. A summary of the number and proportion of samples randomly selected for analysis, based on each respiratory season, is summarised in Table S1B.

Table S1: Summary of samples available for laboratory PCR and proportions of samples selected for PCR.

A - Stored samples available for any analysis				
	**POC test**	**Group A (suspected RSV infection)**	**Group B (non-suspected RSV infection)**	**Total**
Year 1	RSV-negative	170 (38.90%)	86 (19.70%)	256 (58.60%)
	RSV-positive	174 (39.80%	7 (1.60%)	181 (41.40%)
	Total	344	93	437
Year 2	RSV-negative	7 (4.90%)	0 (0.00%)	7 (4.90%)
	RSV-positive	133 (93.70%)	2 (1.40%)	135 (95.10%)
	Total	140	2	142
**B - Number of samples randomly selected for laboratory PCR in each category**				
	**POC test**	**Group A (suspected RSV infection)**	**Group B (non-suspected RSV infection)**	**Total**
Year 1	RSV-negative	37	18	55
	RSV-positive	35	1	36
	Total			91
Year 2	RSV-negative	1	0	1
	RSV-positive	31	1	32
	Total			33
	Cumulative	104	20	124
**C - Proportion of available samples selected for laboratory PCR in each RSV season**				
	**POC test**	**Group A (suspected RSV infection)**	**Group B (non-suspected RSV infection)**	**Total**
Year 1	RSV-negative	40.7%	19.8%	60.4%
	RSV-positive	38.5%	1.1%	39.6%
	Total			100.0%
Year 2	RSV-negative	3.0%	0.0%	3.0%
	RSV-positive	93.9%	3.0%	97.0%
	Total			100.0%

Table S2: Clinical characteristics of children included in RT-PCR analysis compared to children who were not included.

Participant characteristics	Number (%) (N=124)	Number (%) N (455)	*p* value
**Age of child**			
< 2 months	50 (40.3)	181(39.8)	0.63
≥ 2 < 6 months	26 (21)	119 (26.2)	
≥ 6 < 12 months	28 (22.6)	88 (19.3)	
≥ 12 < 24 months	20 (16.1)	67 (14.7)	
**Median age in days/ IQR**	100 (30-277)	92 (32-251)	0.85
**Mean birth weight in Kg/SD**	2.8 (0.53)	2.9 (0.58)	0.57
**Born preterm (<37 weeks)**	13 (10.5)	47 (10.5%)	0.67
**Sex**			
Female	38 (30.6)	160 (35.2)	0.34
Male	86 (69.4)	295 (64.8)	
**Nutritional status (WHO growth standards)**			
Mean WFAZ score (95% CI)	-1.4 (-1.7 to -1.1)	-1.3 (-1.5 to -1.1)	0.73
Mean HFAZ score (95% CI)	-1.2 (-1.5 to -0.9)	-1.3 (-1.5 to -1.1)	0.41
Mean WFHZ score (95% CI)	-0.8 (-1.2 to -0.4)	-0.5 (-0.7 to -0.2)	0.18
**Breast feeding status**			
Mixed breast & formula fed	61 (49.2)	201 (44.2)	0.31
Exclusively breast fed	57 (46.0)	243 (53.4)	
Formula fed	3 (2.4)	6 (1.3)	
Other	3 (2.4)	5 (1.1)	
**Immunisation status for local schedule and age**			
Fully immunised	60 (48.3)	219 (48.1)	0.80
Partially immunised	53 (43.0)	198 (43.5)	
Not yet vaccinated	10 (8.1)	37 (8.1)	
**Presence/ absence of SARI at admission**			
Fulfilled extended SARI criteria at diagnosis (Group A)	108 (87.1)	380 (83.5)	0.20
Did not fulfil extended SARI criteria at diagnosis (Group B)	16 (12.9)	75 (16.5)	
**Median SpO_2 at admission (IQR)**	88 (86-95)	88 (85-94)	0.72
**Enrolled season**			
Season 1 (Year 2021)	81 (65.3)	296 (65.1)	0.52
Season 2 (Year 2022)	43 (34.7)	159 (34.9)	

Table S3: Comparison of clinical characteristics of children in eSARI group and non-eSARI group.

	e-SARI	Non-eSARI	
Participant characteristics	Number (%) (n = 484)	Number (%) (n = 95)	*p* value
Median age in days/ IQR	85 (30-235)	126 (38-372)	0.04
Female sex	161 (33.3)	37 (38.9)	0.28
Birth weight in Kg, mean (SD)	2.9 (0.5)	2.7 (0.5)	0.02
Current weight, mean (SD)	5.6 (2.5)	5.8 (3.1)	0.42
Current length, mean (SD)	59.8 (10.2)	61.8 (11.6)	0.09
Born preterm (<37 weeks)	44 (9.1)	16 (16.8)	0.67
Exclusively breast fed	239 (49.8)	48 (50.5)	0.89
Fully immunised for local schedule and age	256 (53.1)	22 (23.2)	< 0.001
SpO_2 at admission, Median (IQR)	88 (85-94)	93 (86-96)	< 0.001
Haemoglobin at admission, mean (SD)	11.8 (2.6)	11.1 (23.9)	0.02
Enrolled in Season 1 (Year 2021)	344 (71.1)	93 (97.9)	<0.001
Samples included in RT-PCR testing	104 (21.5)	20 (21.1)	0.92

Table S4: Characteristics of children with discordant test results in two platforms.

Participant ID	POCT test result#	RT-PCR test result*	RSV Subtype	CT Value	Age in days	Sex	Diagnosis at admission
NP-KCH-497	+	-	NA	NA	11	Male	SARI
NP-KCH-150	-	+	RSV-A	35.0	24	Male	SARI
NP-KCH-216	-	+	RSV-A	34.2	202	Female	SARI
NP-KCH-148	-	+	RSV-A	35.5	22	Male	SARI
NP-KCH-151	-	+	RSV-A	31.5	06	Female	SARI
NP-KCH-267	-	+	RSV-A	31.0	45	Male	Non-SARI

#POCT instrument readout was positive or negative only; viral load or CT values are not reported
*RSV subtype determined by RT-PCR.
Abbreviations: –, negative; +, positive; CT, cycle threshold; NA, not applicable; POCT, point-of-care test; RSV, respiratory syncytial virus; RT-PCR, reverse-transcription polymerase chain reaction.

Table S5: Summary of discordant results by disease phenotypes.

Disease phenotype	POCT	PCR Result		Total
		Positive	Negative	
Group A (eSARI)	RSV-negative	4	36	40
	RSV-positive	67	1	68
Group B (non-eSARI)	RSV-negative	1	13	14
	RSV-positive	2	0	2

REFERENCES

1. Löwensteyn YN, Mazur NI, Nair H, Willemsen JE, van Thiel G, Bont L. Describing global pediatric RSV disease at intensive care units in GAVI-eligible countries using molecular point-of-care diagnostics: the RSV GOLD-III study protocol. BMC Infect Dis. 2021; 21:857.

2. Löwensteyn YN, Zheng Z, Rave N, Bannier M, Billard MN, Casalegno JS, et al. Year-Round Respiratory Syncytial Virus Transmission in The Netherlands Following the COVID-19 Pandemic: A Prospective Nationwide Observational and Modeling Study. J Infect Dis. 2023; 228:1394-9.
